# Improved Wear-Resistant Performance of Epoxy Resin Composites Using Ceramic Particles

**DOI:** 10.3390/polym14020333

**Published:** 2022-01-15

**Authors:** Amal Nassar, Mona Younis, Mohamed Ismail, Eman Nassar

**Affiliations:** 1 Mechanical Engineering Department, Higher Technological Institute, Next to Small Industries Complex, Industrial Area 2, 10th of Ramadan City 11111, Egypt; mona.younis@hti.edu.eg (M.Y.); eman.nasser@hti.edu.eg (E.N.); 2 Mechanical Engineering Department, The British University in Egypt, Suez Desert Road, El Sherouk City 11837, Egypt; mohamed.elzareef@bue.edu.eg

**Keywords:** polymer composite, kraft paper, wear, recycling

## Abstract

This work investigated the effects of using a new fabrication technique to prepare polymer composite on the wear-resistant performance of epoxy resin composites under dry friction conditions. Polymer composite samples with different weight contents of silicon carbide (SiC) particles were manufactured. This paper addresses the wear behavior of the obtained samples. With the suggested technique, the samples were prepared from epoxy/silicon carbide particles using a layer of thin kraft paper to prevent the sedimentation of the ceramic particles and to control the weight content of ceramic in the polymer. Kraft paper was used as a layer in the polymer composite. The hardness, wear resistance, and water absorption capacity of the produced epoxy composite samples prepared using the kraft paper technique were evaluated. The morphology of epoxy composite samples showed a significant improvement in the ceramic distribution and enhancement of interface bonding between ceramic and the polymer. The hardness values of the developed polymer composites were enhanced by up to 42.8%, which was obtained at 18 wt.% SiC particles. Increasing the ceramic content in the epoxy also led to the enhancement of wear resistance compared with pure epoxy. The results of the microstructure study also showed that the kraft paper layers helped in maintaining the distribution of the ceramic particles according to the previously specified content in each layer in the sample. Wear tests showed that the wear rate of the polymer composite decreased with the increase in the ceramic content. This study provides a new recycling method for using old kraft paper in polymer composite manufacturing to improve the distribution of ceramic particles in the polymer matrix.

## 1. Introduction

Surface modification is a useful approach for functional laminate composite material to improve wear resistance and impact strength [1]. Due to their high strength-to-weight ratio, polymers have received significant attention in different mechanical manufacturing fields. One of the greatest disadvantages of polymers is that they are prone to wear and have little resistance to friction. Therefore, it is important to look for ways to improve and enhance the surface’s wear resistance through surface reinforcement. Functionally graded material is a technique to fabricate components with location-specific properties [2]. It can be used to obtain high surface hardness and sufficient internal toughness that cannot be obtained with homogeneous or monolithic materials [3]. In addition, functionally graded processes can modify the thermal and mechanical properties of composites by using a suitable combination for matrix and reinforcement with suitable properties [4]. Ceramic reinforcement of a polymer matrix can produce a single material with excellent surface protection and friction resistance capabilities, thereby eliminating the possibility of wear [5]. Epoxy resin is a thermosetting polymer used to prepare composite materials and is known for its superior characteristics, such as exceptional mechanical and thermal performance and corrosion resistance against corrosive solutions and environments. In addition, epoxy resin can be modified by adding the appropriate reinforcing materials to obtain the desired properties [6]. As a crosslinked polymer, epoxy is considered a low-toughness material with a low resistance rate [7].

Silicon carbide (SiC) is an odorless black powder that has excellent thermal, chemical, and mechanical properties. Silicon carbide can be used to produce polymer composites [8]. Therefore, the mechanical properties of polymer interleaved with microsized SiC ceramic particles have been examined in many investigations. The wear resistance of epoxy resin can be enhanced by forming a friction-resistant layer of ceramic material, which can extend the use of polymers in highly abrasive applications when the parts are subjected to friction force, such as in links and joints. Polymers filled with ceramic particles have been extensively studied in terms of their wear resistance behavior. Bahadur et al. [9] stated that fillers such as CaS, CuS, CaO, and CuF2 can decrease the wear rate of the polymer. They also reported that the wear rate was reduced with the addition of CuF2 and CuS fillers to polyether ether ketone [10]. Wang et al. [11,12] proved that the wear resistance coefficient of friction of polyether ether ketone was enhanced by adding 7.5 wt.% nanosized ZrO2. They also reported that the enhancement of wear resistance was related to the strength of the bond between the counterface and transfer film [11]. Rangaswamy et al. [12] studied the effect of adding different amounts of MWCNT fillers to hybrid composites on their mechanical properties. They found that hybrid composites showed a higher wear resistance than those found in neat composites. Polymer composites can be produced using several fabricating techniques, such as hand layup [13], molding of the vacuum bag [14], molding of resin transfer [15], and injection molding [16,17]. Although various fabrication methods are available to produce polymer composites, very few techniques can fabricate composites successfully due to differences in the nature of the matrix and particles [17]. Chan et al. [14] reported that traditional manufacturing methods such as hand layup and out-of-autoclave to produce polymer composites are not suitable for producing complex composites. Identifying a suitable technique to produce polymer composites according to the type of reinforcement and the type of matrix requires further research and studies.

Ceramic particles that are used as reinforcement in polymer composites can also improve thermal properties [18]. In addition, adding microsized ceramic particles enhances the toughness properties of polymer composites. The type of reinforcement, weight percentage, and type of polymer material are the major parameters that influence the behavior of polymer composites reinforced with ceramic particles [19]. Determining the suitable amount of reinforcement that will improve the mechanical properties without any side effects on the physical properties, such as shrinkage and poor surface finish, requires further research and studies.

Kraft paper is a type of paper made by kraft processing, which involves chemical treatment to convert wood to wood pulp [20]. The kraft process involves changing the structure of the components of wood by separating cellulose from lignin to produce good paper [21]. Because kraft paper has low lignin content and a high sulfuric ratio, no bleaching process is used, and kraft paper is stronger compared with other types of paper [22]. Kraft paper is a porous material that has excellent absorbency for liquids, which makes it suitable for producing epoxy composites [23,24].

To improve the wear resistance of polymer composites, various techniques have been used, such as the use of nano- or microsized ceramic materials as fillers in polymers such as silicon carbide [25,26], zirconium oxide [27], calcium carbonate [28], titanium oxide [29], and calcium oxide [27].

In addition, heat treatment is also considered to be an effective method to improve the wear resistance of polymers. Ayman et al. [30] proved that heat treatment could improve the wear resistance properties of polyamide (PI). Wang et al. [31] investigated the effect of the pressure of the molding on the tribological and mechanical properties of ultra-high-molecular-weight polyethylene, and they found that the wear rate was decreased by heat treatment. Sarkar et al. [32] mentioned that the wear resistance of polymers can be improved by the heat treatment process. In ceramic/epoxy composites, first, the ceramics are dispersed in the liquid polymer, and then the solidification process starts. Complete solidification of the liquid polymer takes a long time. During this time, ceramic particles will sediment at the bottom, and the upper area of the polymer will be free of ceramic particles [26,33].

As the polymer is softer than metals [27] and there are limited methods for enhancing the polymer, the present work was an attempt to enhance epoxy resin using a prespecified number of ceramic layers by using a novel technique to control the ceramic content in the polymer.

Using ceramic particles such as microsized SiC is one way to enhance the wear resistance of a polymer; however, the literature studies are mostly focused on the type and the amount of reinforcement filler. Therefore, this study investigated the effect of using natural material as a technique to enhance the distribution of ceramic particles in epoxy, thus improving the wear resistance of the polymer. The literature review also confirmed that traditional manufacturing techniques to produce polymer composites require significant research attention. Furthermore, to the authors’ knowledge, no attempts have been made to use external materials to produce functionally graded polymer composites. The contributions of the present work are the following:

1. Functionally graded polymer composites (epoxy/SiC) that are suitable for highly abrasive applications were developed.
2. The effect of wt.% content of SiC on the impact strength, hardness, and wear resistance was investigated.
3. The degree of adhesion of kraft paper in composite polymer and the SiC distribution were evaluated by SEM images.

In addition, this paper presents a new technique to control the distribution of the particles in the matrix to obtain the desired amount of ceramic content in the polymer.

## 2. Materials and Methods

### 2.1. Materials and Sample Preparations

The polymer used is Kemapoxy 150 JM, supplied by CMB Egypt. Two sizes of ceramic silicon carbide particles were used in the investigation: medium size, which is a particle size less than 25 μm, and fine size, which is a particle size less than 90 nm. Both sizes were supplied by Sigma Aldrich, Germany.

Silicon carbide powder was selected because it is a hard ceramic commonly used to protect surfaces against friction and wear. Recycled paper bags made from kraft paper were used in the experiments. Paper bags were cut into circular pieces to produce paper lamina with a diameter equal to 900 mm.

Ceramic particles were cleaned with deionized water to remove any impurities. The epoxy resin composite mixture was produced using a combination of epoxy resin, hardener, and specified wt.% of silicon carbide powder, which was mixed by using mechanical stirring for 4 min with a speed of 500 rpm and heated by an electrical heater (90 °C) for 1 min to remove air bubbles generated during mixing and to reduce the cure time. The preparation process was carried out at room temperature inside a circular wood mold with a radius of 1400 mm and 40 mm depth. All obtained composite samples were left under a hydraulic press for 8 h until they were completely dried.

**Figure 1:**
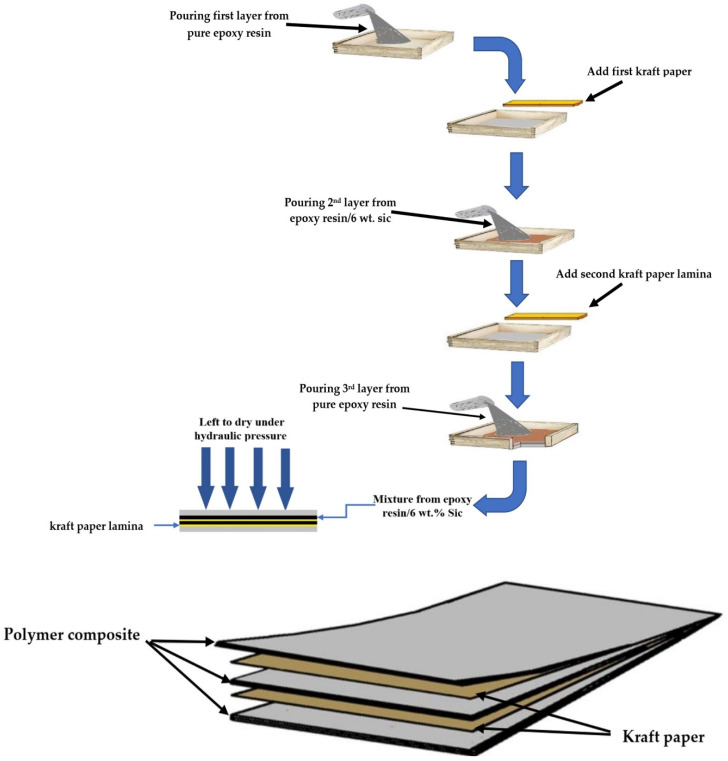
Flowchart representing the steps of the hand layup technique for preparing polymer composite samples.

The final thickness for all obtained samples was 2 mm to meet the requirements of DIN EN ISO 527-4. The schematic view of the experimental setup is presented in Figure 1. The amount of ceramic content varied in each location (layer), but the total wt.% ceramic contents for samples were as follows:

- Samples no. 1, 2, 3, and 11 were reinforced with 6 wt.% SiC.
- Samples no. 4, 5, and 6 were reinforced with 12 wt.% SiC.
- Sample no. 7 was reinforced with 18 wt.% SiC.
- Sample no. 8 was reinforced with 15 wt.% SiC.
- Samples no. 9 and 10 were reinforced with 9 wt.% SiC.
- Sample no. 12 was reinforced with 3 wt.% SiC.

Table 1 shows the experimental design levels (k) and the range of independent variables (n) used in this work according to a full factorial design n^k^. The experimental design matrix of different compositions is presented in Table 2 and was prepared using the statistical software Design-Expert.

### 2.2. Identifying Thickness of Layers

To prepare polymer samples with different contents of ceramic particles, the particle settling time was calculated theoretically and experimentally to find the suitable thickness (distance between each layer). The thickness of the layer was modified theoretically by calculating the settling velocity of the ceramic particles using Stoke’s law, which assumes that: all particles are spherical, there is no interaction between neighboring particles that are settling, and there is complete wetting between the dispersed particles and the fluid [28,29]. Stoke’s law estimates the settling rate of a particle by:

Vp = [ (d^2^ (ρ_ρ −_ ρ_1_)) g]/18 μ (1)

## 3. Characterization of Composites

Composite samples were cut into the required dimensions by using a laser cutter. The composite samples were examined according to the following standard tests.

### 3.1. Physical Tests

The densities of the studied samples were measured with a densimeter (DH-300K DahoMeter Digital Electronic, Dongguan Hong Tuo Instrument Co., Guangdong Province, Dongguan, China) at room temperature based on the standard D 792–07. The density of each sample was recorded as the average of three readings. Water absorption of the obtained samples was evaluated according to the standard test method of D570 ASTM. For density and water absorption tests, the specimens were cut into the form of a disk 50.8 mm in diameter and 2 mm in thickness. In the water absorption test, five samples from each type were examined according to the following sequence: firstly, the weight of dried samples was recoded after drying the samples in the oven at 100 °C for 12 min, and samples were left to dry at room temperature for 18 h. Secondly, the samples were submerged in a glass container filled with 900 mL of pure water at room temperature (27 °C).

The weight of the samples was measured periodically for 600 h. The samples were weighed regularly every day by using a 4-digit analytical balance, and the absorption capacity was calculated by using Equation (2):

(2) $$\mathrm{WC}=\frac{WC-WO}{WO}\times 100\;$$

### 3.2. Mechanical Tests

The ability of the polymer composite to resist wear under friction was analyzed by performing wear rate tests. Due to the many possible combinations of wear test parameters, such as sliding speed, load, temperature, and sample type, a wide range of wear results for the same polymer composite sample can be obtained. All wear rate measurements were performed at room temperature in dry conditions using a reciprocating sliding test setup for a 100 m distance at different loads (7.5, 10, 12.5, and 15 N), and the load was kept constant during testing by using the T-01M tribometer. The ball-on-disk method was selected due to the small contact area and simple setup. In the test, the surfaces of samples were subjected to friction force by a ball made of chrome steel, SAE 52100. Table 3 summarizes its full characteristics. Before conducting the test, each sample was cleaned with an organic solvent and left to dry with hot air (60 °C). To study the wear mechanism, samples were observed with a scanning electron microscope (JSM 6480 LV, JEOL, Peabody, MA, USA) at 30 kV. Results are the average readings of three fabricated samples for each case; the samples were cut into a ring shape with an outer diameter equal to 50 mm and inner hole diameter equal to 20 mm, and the thickness of the ring was equal to 2 mm. The forming was produced by a laser cutting machine. The wear rate of each sample was calculated by determining the weight loss. The samples were weighed before and after the wear test using a 4-digit analytical balance and then using Equation (3) to calculate the value of the wear rate:

(3) $$W=\frac{w.l}{L\;\rho w\;F}$$

The results of the weight loss were compared with similar ones obtained under the same conditions for each wt.% SiC.

The Shore D hardness (needle pin steel rod) value was measured to study the effect of ceramic content on composite hardness. The hardness test was carried out according to the standard CSN EN ISO 868. The hardness was measured at 5 points on the sample surface, and the hardness value is the average of the hardness values at these points. Three samples were used for each sample type.

## 4. Results and Discussion

### 4.1. Time of Particle Settling

**Figure 2:**
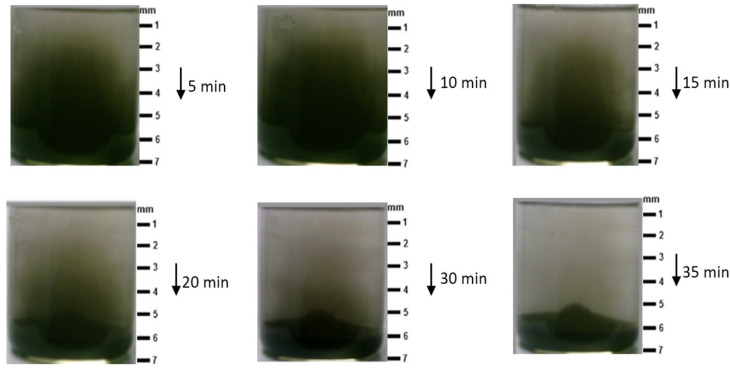
Settling of the SiC suspension over time: 5 min, 10 min, 20 min, 30 min, and 35 min.

Table 4 shows the estimated results for the particles settling in the epoxy resin. The pot lifetime of Kemapoxy 150 JM or the epoxy’s cure speed was about 45 min, and the experimental measurements of different contents of SiC showed that almost all particles settled at the bottom by 35 min after finishing the mixing process. Figure 2 shows photo images depicting the motion of the suspended SiC particles with time at room temperature. It is clear from the images that all particles were concentrated at the bottom during the solidification of epoxy resin. This means that the polymer became free of ceramic content, exceeding the bottom area that will hold all of the ceramic particles.

**Figure 3:**
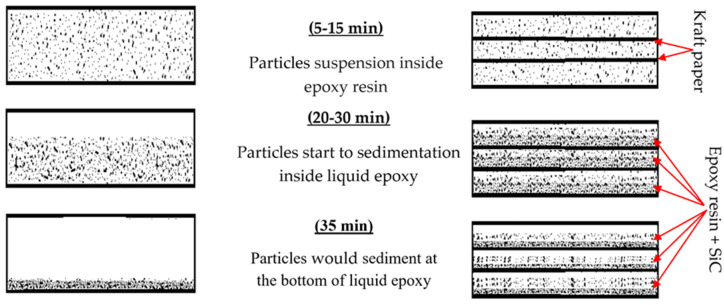
Schematic representing the effect of using kraft paper as separating layers in epoxy.

**Figure 4:**
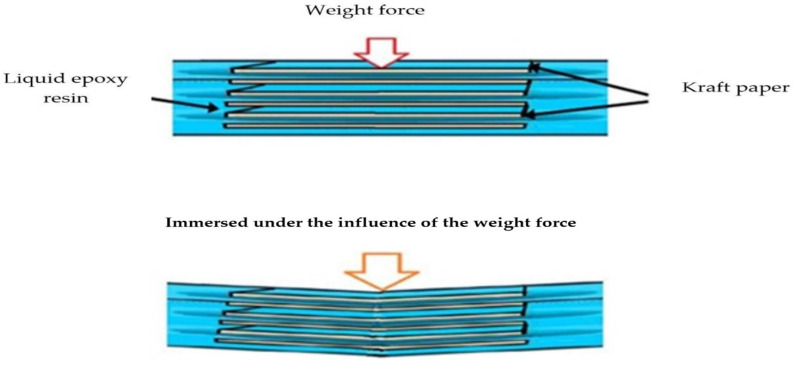
Schematic of the influence of increasing the number of layers on liquid polymer.

Therefore, to overcome the problem of particle sedimentation due to the long drying time, kraft paper was used as a separating layer between each polymer content to ensure that the particles were prevented from moving and sedimentation. In addition, the liquid polymer composites were heated to 90 °C by using an electrical heater to reduce the cure time [33]. Thus, the presence of the required ceramic content within the sample was ensured. Figure 3 schematically shows the motion of the suspended SiC particles at different times in both cases. In the traditional method, if ceramic particles are mixed in the epoxy resin and the polymer composite is left to cure, the ceramic particles will settle at the bottom. However, the proposed method can stop particle deposition and enhance the distribution of the particles in the polymer. In addition, a reduction in the cure time leads to the rapid occurrence of crosslinking in the polymer, which stops the motion of particles and their sedimentation. On the other hand, increasing the number of layers to more than three may lead to the possibility of the kraft paper becoming immersed in the liquid polymer under the influence of the weight of each layer, thus losing the good distribution of the particles, as shown in Figure 4.

### 4.2. Morphology

**Figure 5:**
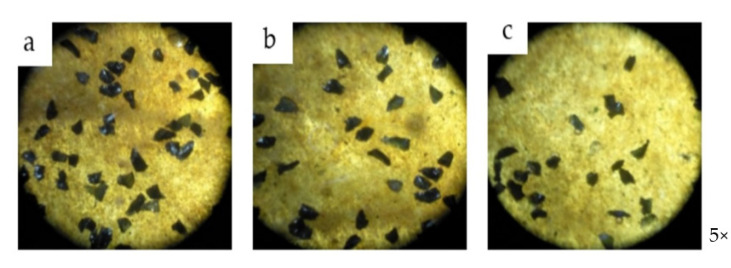
Microstructure photographs illustrating top surfaces of fabricated polymer composite samples. (**a**) At 18 wt.% of SiC; (**b**) At 12 wt.% of SiC and (**c**) At 6 wt.% of SiC.

**Figure 6:**
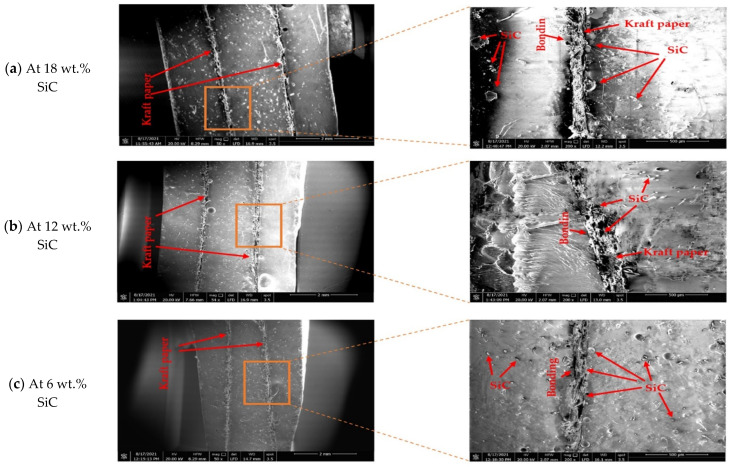
SEM images of composites showing distribution of ceramic particles in epoxy with two magnifications: (**a**) 18 wt.% SiC, (**b**) 12 wt.% SiC, (**c**) 18 wt.% SiC.

SEM photomicrographs and microscopic photographs were used to ensure that the ceramic particles were uniformly distributed within the polymer. Figure 5 shows the microscope photographs of top surfaces of the polymer composite microstructure at different wt.% of the ceramic material. The microscopic photographs clearly show the bonding between each layer of the polymer composite with different contents of SiC wt.%. Figure 6 shows SEM photomicrographs with two magnifications and different contents of SiC wt.%. The figure reveals that the SiC particles are completely dispersed in the polymer. In addition, high ceramic content was found at the upper layer of the kraft paper due to the action of gravity. At a low SiC concentration (6% wt.%), the ceramic particles were dispersed in the sample, and larger particles were concentrated on the kraft paper (see Figure 6a). As the ceramic content increased, particles of SiC were dispersed uniformly, and larger particles were concentrated above the kraft paper (see Figure 6b). This may be due to the difference in density between ceramic and epoxy, making the SiC tend to settle in the liquid epoxy. However, the fine-size SiC did not have a tendency to sediment, which is due to the viscous drag effect of the fine-size particles. This drag is dependent on the surface area. Therefore, because the fine particles have a small surface area compared with the large particles governing the effect of small grains, they settle more slowly [34]. The figure also shows the excellent bonding between ceramic and epoxy and between kraft paper and epoxy. It can be said that the functionally graded polymer can be obtained by using kraft paper as a tool to stop filler from moving within the liquid polymer.

### 4.3. Physical Tests

#### 4.3.1. Density

**Figure 7:**
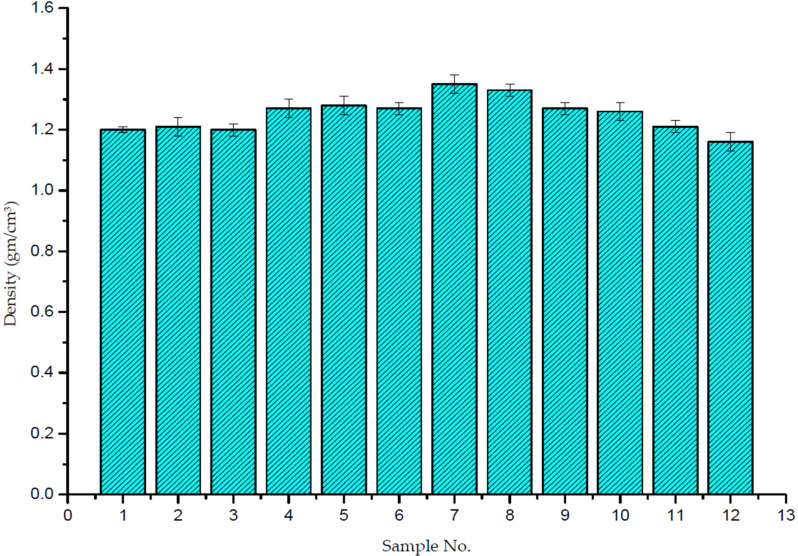
Densities of composite samples.

Figure 7 shows the density of composite samples under different conditions. It is clear from the figure that the density increases with the further increase in wt.% of reinforced particles, regardless of the location of the weight% content. The same results were found by Stukhlyak et al. [35]. They observed that ceramic particles tended to create pores in the matrix, which affected the density value of the composite. The increase in density can be related to the fact that ceramic is heavier than epoxy [14].

In this study, it was found that the density of the composite samples was equal to 1.21 gm/cm^3^ for 6% wt.% SiC-reinforced composite materials, 1.28 gm/cm^3^ for 12% wt.% SiC, and 1.35 gm/cm^3^ for 18 wt.% SiC. These values are greater than the density of the pure epoxy, which equals 1.18 gm/cm^3^.

#### 4.3.2. Water Absorption Capacity

**Figure 8:**
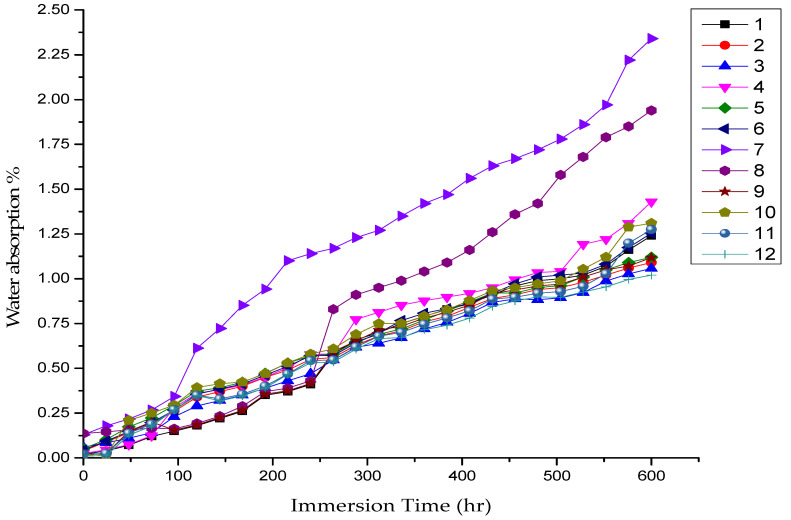
Water absorption capacity (%) as a function of wt.% and location of SiC particles.

The effect of the ceramic amount content and its location on the water absorption behavior of epoxy composites with immersion time is presented in Figure 8. The figure reveals that water absorption of epoxy composites decreased with a decrease in immersion time. According to Wang et al. [36], the hydrogen bond is broken due to the effect of water molecules’ mobility. When the immersion time increases, the mobility of water molecules increases, which leads to an increase in the rate of water absorption of composite samples. On the other hand, the rate of water absorption increases with the increase in ceramic content. A similar observation was also reported by Dhakal et al. [37] when they worked on Lantana Camara fiber-reinforced epoxy composite. This may be due to the increase in voids with high ceramic content and due to poor ceramic–epoxy adhesion, which might have led to microcrack formation on the outer surface of the sample due to ceramic agglomeration within the matrix [38]. In addition, the ceramic content is more likely to fail under the influence of water, which causes ceramic particles to leave their places and create voids, and then these voids are filled with water as a result of the capillary effect [33]. The order of layers has a limited effect on the water absorption rate. Samples 7 and 8 showed higher water absorption than the other samples. This is due to the high ceramic content in these samples. Sample 8 had the maximum rate of water absorption due to the concentration of particles in the same layer.

### 4.4. Mechanical Properties

#### Hardness Measurement

**Figure 9:**
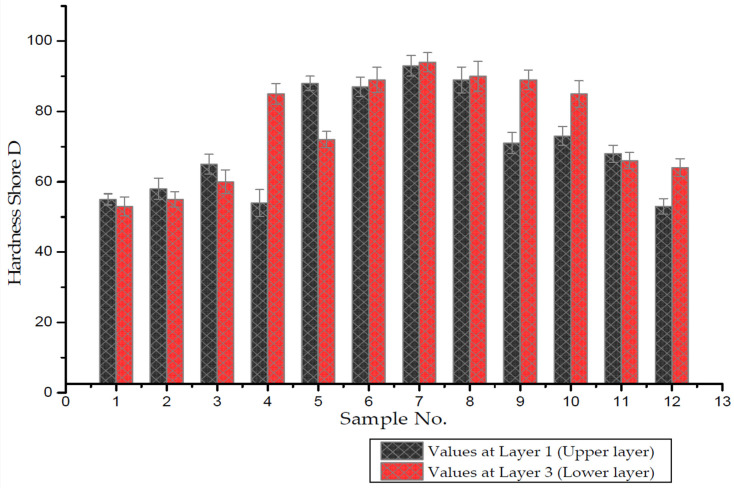
Barcol hardness as a function of wt.% and location of SiC particles.

Shore D hardness values were measured on the upper surface (layer no. 3) and lower surface (layer no. 1) with different amounts of ceramic content. Figure 9 represents the hardness values as a function of the sample number. The figure clearly shows the effect of the amount of ceramic content on the hardness values. Furthermore, the location of ceramic content had a large effect on the hardness values. Minimum and maximum hardness values for layer no. 2 and layer no. 1 of the composite samples were found to be 50.91 and 52.94, respectively. The results also reveal that hardness values increased as ceramic content increased in epoxy composites due to the distribution of the test load on the composite surface [39]. The hardness of ceramic composites is defined by the amount of ceramic content, ceramic type, and composite density. This is due to the excellent dispersion of ceramic particles in epoxy and the high hardness of silicon carbide, all of which will lead to an increase in the hardness of the epoxy composites [29].

Hardness values were enhanced by 20%, 36.2%, and 42.8% with 6 wt.%, 12 wt.%, and 18 wt.% SiC particles, respectively, as compared to pure epoxy. The maximum values of hardness were obtained with a high concentration of ceramic particles. Thus, 18 wt.% SiC showed the maximum hardness values. On the other hand, in the case of low ceramic content, the dispersion of ceramic epoxy was not sufficient to distribute the test load on the sample surface [40].

### 4.5. Wear Resistance

The wear resistance of samples reinforced with different amounts of SiC at various locations was measured by ball-on-disk dry sliding methods, and the upper surface (layer no. 3) of the sample was subjected to friction force. The results of wear are presented in Table 5. It is possible to observe that weight loss increased with increasing load applied to the samples. For example, the weight loss of the sample with 0 wt.% SiC in layer 1, 6 wt.% SiC in layer 2, and 0 wt.% SiC in layer 3 was 6.81 ×10^−3^ (g) at 7.5 N and 6.98 ×10^−3^ (g) at 15 N. This is due to the increased load, which led to a rise in friction between the sample and ball, which led to abrasive wear in the sample, thus increasing the weight loss. The results show that the weight loss was increased for epoxy reinforced with 0 wt.% SiC in layers 2 and 3 and 6 wt.% SiC in layer 1. The weight loss was 6.81×10^−3^ (g) for the sample with 0 wt.% SiC in layer 1, 6 wt.% SiC in layer 2, and 0 wt.% SiC in layer 3, and it increased to 6.86 ×10^−3^ (g) in the same friction conditions. However, the weight loss decreased with the increase in the speed of rotation and ceramic content in the subjected layer. For example, the weight loss values were 4.76 × 10^−3^ g at a rotation speed equal to 300 rpm and 4.23 × 10^−3^ g at a rotation speed equal to 1200 rpm. A lower weight loss was obtained by epoxy reinforced with 18 wt.% SiC in layer 3, as revealed in Table 5. This is due to the ceramic particles serving as protection for the polymer.

In addition, the location of ceramic particles was an important parameter contributing to the value of weight loss. In other words, if the subjected layer in a sample is filled with high content of ceramic particles, and this sample is subjected to friction force, it resists and withstands this severe condition. The role of kraft paper in holding ceramic particles at the proper location in epoxy is clearly demonstrated. The weight loss for 6 wt.% SiC in layer 1 and layer 2 decreased from 4.61 ×10^−3^ (g) to 4.38 ×10^−3^ (g) for 6 wt.% SiC in layer 3 and layer 2. This is due to an increase in the ceramic content in the polymer at the layer subjected to friction force. Fibers of the kraft paper lamina also function as a holding basket for the ceramic particles under the effect of friction force. In other words, retaining the ceramic particles in the polymer plays a significant role in protecting its surface from wear during the application of the friction force, a role that the suggested method successfully fulfilled.

## 5. General Comments and Discussion

Polymers are promising materials due to their superior characteristics, but they cannot resist friction force. In the current preparation method, SiC ceramic particles were distributed within epoxy resin and were prevented from settling during the epoxy solidification process due to differences in density between liquid epoxy and ceramic particles. Kraft paper works as a dam against ceramic movement, which leads to improved particle distribution in the polymer. Therefore, when the polymer composite sample is subjected to friction force, it resists wear. The improvement in the wear resistance is attributed to the perfect distribution of ceramic particles in the polymer, which helps in protecting the polymer [41].

**Figure 10:**
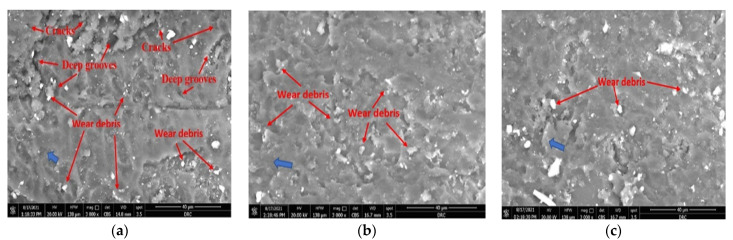
SEM photomicrograph of the worn surface after wear tests of: (**a**) epoxy/6 wt.% SiC, (**b**) epoxy/12 wt.%. SiC, (**c**) epoxy/18 wt.%. SiC. Note: Blue arrow refers to sliding direction.

The weight loss increases with higher loads, which is a result of the increase in the pressure of the rough worn surface on the sample and therefore an increase in friction force. The weight loss is lower for the polymer reinforced with a high content of SiC particles. This is due to the higher specific modulus of SiC particles in comparison to plain epoxy [41]. To study the wear mechanism of polymer composites under different conditions, the worn surfaces of wear samples were studied by using SEM (Figure 10). The images proved that samples with a small amount of SiC up to 6 wt.% in the upper surface were subjected to a severe abrasive wear mechanism due to the appearance of large cracks and deep grooves in the polymer surfaces [1,35]. Figure 10a shows a high amount of debris and deep cracks on the worn surface, which confirms the abrasive wear in samples with a low content of ceramic. In addition, the cracks in these samples were perpendicular to the direction of sliding [42].

Generally, using polymer composites is dependent on the required applications and features that the polymer composites can provide. Silicon carbide reinforcement can be used in many applications where wear resistance, strength, and hardness are necessary, such as marine and aircraft applications. However, low-cost micron SiC reinforcement can be a suitable alternative to high-cost nano metal reinforcement for applications that require moderate mechanical properties, such as sports equipment, car accessories, and biomedical joints.

**Figure 11:**
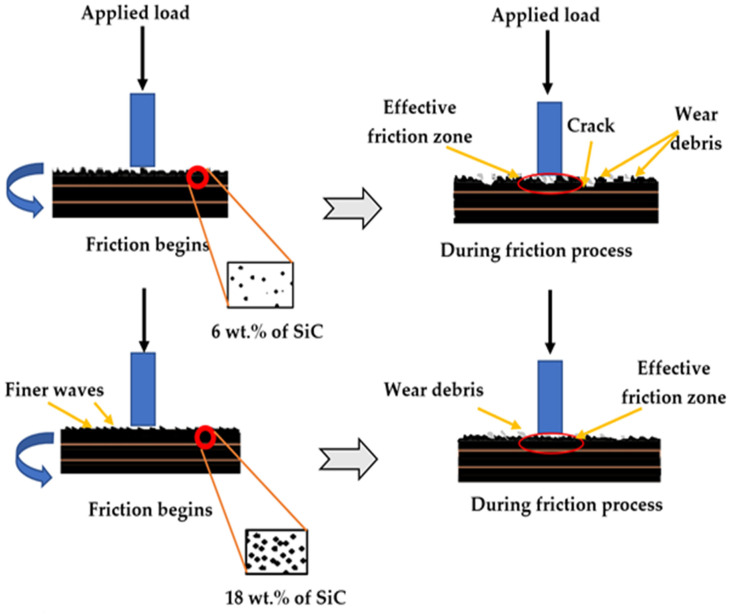
Schematic of expected wear steps during bin-on-disk wear test.

Figure 11 is a schematic explanation for the expected wear steps, and it shows details of the mechanism that occurs during wear tests. With a low content of ceramic particles, the waves adopt a thinner shape compared with the large content of ceramic, where the waves are thick. Furthermore, in the sample with a low content of ceramic, the polymer is easily peeled off under the effect of friction force and produces a high amount of wear debris. On the other hand, the large content of ceramic protects the polymer surface from wear under friction force, which appears clearly in Figure 10c as a low amount of debris and relatively few cracks in the worn surface. Cracks were seen in the samples with a low content of ceramic particles up to 6 wt.% SiC (Figure 10a). However, the cracks were reduced in samples with a high content of ceramic particles because the ceramic particles hindered the generation of cracks in the sample. The smooth wear surface appeared for the high ceramic content due to the difference in bonding power between ceramic particles and the epoxy matrix. The mechanism of wear adhesion is considered the affected factor to describe the wear behavior. A decrease in ceramic content was observed to lead to a decrease in wear resistance and loss of the metal. The friction force due to load pressure caused plastic deformation in the surface layer by removing the soft or unprotected surface and producing a high amount of debris. On the other hand, increasing the ceramic content will lead to an increase in the friction between surfaces, causing an increase in the temperature, which causes a gradual flattening of the protrusions. This flattening leads to a smooth surface, decreases adhesion, and increases the sliding speed, which reduces the wear rate.

## 6. Conclusions

The effects of using a new fabrication technique to prepare polymer composite on wear-resistant performance were investigated. It was concluded that using external natural material had a significant effect on the ceramic distribution in the polymer. Kraft paper was used as an external natural material to hinder the sedimentation of the ceramic particles and to control the weight content of ceramic in the polymer. The experimental results show that using kraft paper as a separation layer in liquid polymer composites prevented ceramic particles from settling during the polymer solidification process. Therefore, excellent wear resistance was obtained, and due to improvement in the distribution of filler particles in the matrix, a decrease in the weight loss was found. Hardness measurements of samples revealed that maximum hardness values were obtained for samples containing 18 wt.% SiC particles, which showed a hardness value of 42.8%, particularly in the upper and middle layers. Moreover, samples with a higher content of ceramic particles showed distinct water absorption capacity compared with lower-content samples. The results also reveal that the water absorption of epoxy composites decreased with a decrease in immersion time and increased with the increase in ceramic content. Morphological investigations of sample cross-sections revealed that the SiC particles were well dispersed within the epoxy resin and had bonded well with epoxy. Wear resistance experiments of samples revealed that lower weight loss was obtained by epoxy reinforced with 18 wt.% SiC in the upper layer, which showed a weight loss of 3.3×10^−3^ (g) at the minimum load and maximum sliding speed. In addition, the location of ceramic particles was an important parameter contributing to the value of weight loss. For the same content of ceramic particles, the weight loss increased by 2.5% when the content location was changed.

## Figures and Tables

**Table 1:** Levels of experimental design and range of independent variables.

Experimental Parameters	Actual Value (Coded Value)		
Layer no.	1 (−1)	2 (0)	3 (1)
Concentration of ceramic, wt.%	0 (−1)	3 (0)	6 (1)

**Table 2:** Experimental design matrix of different compositions.

Sample No.	Coded Factors			Actual Factors (SiC wt.% Content)		
	Layer No. 1	Layer No. 2	Layer No. 3	Layer No. 1	Layer No. 2	Layer No. 3
1	1-	1	1-	0	6	0
2	1	1-	1-	6	0	0
3	0	0	1-	3	3	0
4	1-	1	1	0	6	6
5	1	1	1-	6	6	0
6	1	1-	1	6	0	6
7	1	1	1	6	6	6
8	1	0	1	6	3	6
9	1	1-	0	6	0	3
10	0	1-	1	3	0	6
11	0	1	0	3	6	3
12	1-	1-	0	0	0	3

**Table 3:** Ball characteristics.

	Value
Diameter	6 mm
Hardness	80 HRC
Surface roughness	0.06 μm

**Table 4:** Theoretical data for calculating Stoke’s settling velocity of SiC suspension in epoxy resin.

Density of the Fluid	1170 kg/m^3^	Stoke’s Settling Velocity Vp = 2.4 × 10^−5^m/s
Viscosity of the fluid	0.029 (kg/ms)	
Average particle diameter particle size	25 μm	
Density of particles	3210 kg/m^3^	

**Table 5:** Weight loss of each sample.

Sample No.	Load (N)	Weight Loss × 10^−3^ (g)			
		at 300 rpm	at 600 rpm	at 900 rpm	at 1200 rpm
1	7.5	6.81	6.77	6.69	6.63
	10	6.86	6.8	6.71	6.68
	12.5	6.9	6.83	6.78	6.7
	15	6.98	6.85	6.78	6.73
2	7.5	6.86	6.82	6.71	6.67
	10	7.02	6.95	6.91	6.87
	12.5	7.06	7.01	6.97	6.91
	15	7.09	7.14	7.03	6.94
3	7.5	5.71	5.66	5.52	5.34
	10	5.74	5.69	5.62	5.56
	12.5	5.78	5.72	5.65	5.59
	15	5.96	5.75	5.69	5.65
4	7.5	4.38	4.25	4.07	3.98
	10	4.72	4.55	4.43	4.25
	12.5	4.92	4.85	4.67	4.45
	15	5.22	5.13	5.01	4.78
5	7.5	4.61	4.54	4.35	4.12
	10	4.83	4.75	4.57	4.44
	12.5	4.97	4.82	4.74	4.56
	15	5.07	4.86	4.81	4.75
6	7.5	4.53	4.34	4.21	4.09
	10	5.02	4.91	4.82	4.71
	12.5	5.19	5.07	4.97	4.83
	15	5.31	5.19	5.05	4.95
7	7.5	3.97	3.81	3.524	3.301
	10	4.05	3.99	3.67	3.31
	12.5	4.13	4.04	3.84	3.65
	15	4.32	4.12	4.01	3.98
8	7.5	4.2	4.06	3.91	3.78
	10	4.56	4.26	3.98	3.85
	12.5	4.76	4.46	4.39	4.23
	15	4.99	4.87	4.63	4.49
9	7.5	5.89	5.71	5.64	5.58
	10	5.95	5.75	5.66	5.61
	12.5	6.02	5.83	5.72	5.67
	15	6.07	5.91	5.77	5.7
10	7.5	5.58	5.44	5.35	5.27
	10	5.64	5.56	5.47	5.34
	12.5	5.69	5.68	5.52	5.41
	15	5.74	5.71	5.64	5.5
11	7.5	5.33	5.26	5.11	5.03
	10	5.46	5.35	5.17	5.11
	12.5	5.66	5.41	5.24	5.15
	15	5.73	5.45	5.36	5.22
12	7.5	7.47	7.35	7.22	7.16
	10	7.51	7.39	7.31	7.23
	12.5	7.53	7.44	7.34	7.27
	15	7.62	7.53	7.42	7.33

## Data Availability

Not applicable.

## Abbreviations

wt.%	Weight percentage
Vp	Stoke’s settling velocity of the particle
d	Particle diameter
ρ1	Density of the liquid
ρρ	Density of the particle
μ	Liquid viscosity
WC	Weight of composite sample in grams after immersion.
WO	Weight of composite sample in grams before immersion.
W.L	Weight loss (measured in g)
L	Sliding distance (m)
ρw	Density of the worn material in g/mm^3^
F	The applied load in N
SEM	Scanning electron microscope
ρp	Density of the particle
